# Nanoparticles as Strategies for Modulating the Host’s Response in Periodontitis Treatment

**DOI:** 10.3390/nano15070476

**Published:** 2025-03-21

**Authors:** Antoaneta Mlachkova, Velitchka Dosseva-Panova, Hristina Maynalovska, Zdravka Pashova-Tasseva

**Affiliations:** Department of Periodontology, Faculty of Dental Medicine, Medical University of Sofia, 1431 Sofia, Bulgaria; a.mlachkova@fdm.mu-sofia.bg (A.M.); v.doseva@fdm.mu-sofia.bg (V.D.-P.); h.maynalovska@fdm.mu-sofia.bg (H.M.)

**Keywords:** periodontitis, therapeutic approaches, host response, innovative strategies, nanoparticles

## Abstract

Periodontitis is a widespread disease, associated with challenges both in its diagnosis and in selecting from various therapeutic approaches, which do not always yield the expected success. This literature review was conducted to explore diverse therapeutic approaches, especially those focused on nanotechnologies, and their potential contribution to the successful modulation of the host’s response. The effects of the existing microbial diversity and the imbalance of key microbial species in contributing to the progression and worsening of the host’s response in periodontitis are well known. It is essential to understand the role of a well-structured treatment plan for periodontitis, providing opportunities for new research and innovative treatment strategies aimed at reducing the impact of periodontitis on oral and overall systemic health. This will be beneficial for dental professionals, enabling them to effectively prevent and treat periodontitis, ultimately improving the overall health and well-being of patients.

## 1. Introduction

Periodontitis is a chronic inflammatory disease characterized by clinical symptoms such as bleeding from periodontal pockets, gingival swelling, the loss of supracrestal connective tissue attachment, and alveolar bone resorption. If left inadequately treated or untreated, it can lead to tooth mobility, tooth loss, and masticatory dysfunction, and even affect the digestive system. Periodontitis is a disease that impacts approximately 11% of the global population, with the prevalence varying between 8% and 46% in developing countries and between 3% and 18% in developed nations [1]. It is more common in older adults due to the cumulative and chronic nature of the condition. In the United States, two-thirds of people over the age of 65 have periodontitis [2]. In recent decades, the occurrence and prevalence of severe periodontitis have risen in Asian countries, including India, China, and Japan [3]. This upward trend is severely impacted by aging populations. The increasing clinical evidence suggests that periodontitis imposes a considerable strain on public health systems due to its association with other health conditions and chronic, autoimmune, and malignant diseases, such as diabetes, Alzheimer’s disease, rheumatoid arthritis, colitis, and even cancer [4,5]. The primary treatment for periodontitis involves the removal of dental plaque and calculus. However, various clinical studies have demonstrated that conventional treatment alone may not sufficiently alter the composition of the subgingival plaque biofilm. As a result, additional therapeutic approaches have been suggested to improve treatment outcomes, including localized drug delivery, systemic antibiotics, and host-response-modulating medications [6].

Multiple meta-analyses have demonstrated that systemic antibiotics and agents modulating the host response can aid in managing periodontitis [7,8,9,10,11]. However, in clinical practice, broad-spectrum antibiotics, whether administered alone or combined with other antibiotics, typically eliminate Gram-negative bacteria within 1 to 3 weeks. The prolonged use of systemic antibiotics and host-response-modulating agents, such as nonsteroidal anti-inflammatory drugs (NSAIDs), carries risks, including but not limited only to antibiotic resistance, microbial imbalance, dysbiosis, gastrointestinal irritation, and harmful drug interactions [7,11,12]. On the other hand, the local delivery of various medications into periodontal pockets has shown effectiveness in treating patients with deep pockets or recurrent periodontitis. Treatment options include nanoparticle delivery systems, hydrogels, fibers, etc. [13,14].

Recent scientific trends highlight the growing use of nanotechnology, which offers significant advantages over traditional methods for local drug delivery to periodontal tissues. Nanoparticles, such as liposomes, polymeric nanoparticles, polymeric micelles, and nanofibers, can be tailored in terms of chemical composition, size, surface charge, and other properties to promote stable tissue retention and durability in the oral environment. Research indicates that nanoparticle carriers safeguard drugs from pH fluctuations and enzymatic breakdown within periodontal lesions. Notably, nanoparticles can be designed to respond to factors such as reactive oxygen species (ROS), pH variations, or enzymatic activity in the pathological microenvironment, enabling controlled drug release [15,16].

## 2. Nanotechnology and Its Significance in the Field of Periodontology

The term “nanotechnology” was introduced in 1974 by Norio Taniguchi (Tokyo Science University), referring to the separation, consolidation, and deformation of materials at the atomic or molecular level. The size of different nanomaterials ranges from 0 to 100 nm. The concept of “nanomedicine” was defined by Freitas in 1993 as the monitoring, control, and treatment of biological systems in the human body at the molecular level using nanostructures and nanodevices. Nanoparticles can be synthesized in two ways: either by assembling smaller elements to form nanoparticles or by breaking down larger molecules into smaller components to reach nanoscale sizes [17,18].

Nanobiosensors can be used to diagnose periodontal diseases by detecting substances in bodily fluids like saliva, blood, and gingival fluid, aiding in the diagnosis, prognosis, and treatment of periodontal conditions [19].

Nanoparticles also offer advantages for the delivery of bioactive molecules and drugs. Biocompatible nanocomposite hydrogels are an effective local delivery method. These materials have a soft and flexible texture, forming a three-dimensional crosslinked polymer network in a moist environment. For example, PerioChip^®^ (Dexcel Pharma Limited, Northamptonshire, UK) is a well-known hydrogel used to deliver chlorhexidine digluconate into deep periodontal pockets. Such technologies can also be applied to periodontal regeneration [14].

Nanotechnologies are considered revolutionary due to their ability to enhance the osteogenic properties of cells. The nano-crosslinked structure offers significant advantages over traditional methods used for tissue regeneration and bone augmentation. Nanofiber membranes made of polycaprolactone or calcium carbonate, used for guided bone regeneration, provide both mechanical strength and tensile durability. These membranes attract osteoblasts, which proliferate on their surface, facilitating subsequent cell attachment [20,21]. Commercially available bone graft substitutes incorporating nanotechnology include Ostim^®^ (Osartis GmbH & Co., Dieburg, Germany), Vitoss^®^ (Orthovita, Inc., Malvern, PA, USA), and NanOss^®^ (Angstrom Medica, Woburn, MA, USA), all based on hydroxyapatite.

Nanotechnology is also applied to current dental implant treatments, aiming to improve osseointegration through chemical and mechanical techniques, as well as nanomodifications of the implant surface (e.g., nano-grooves, nanopillars) [22]. Various nanomaterials, such as bioactive glass, carbon nanomaterials, titanium nanotubes, and nanoceramics, can be used to coat dental implants and support bone regeneration in the treatment of bone defects. Implants with titanium coatings have been proven to enhance the osseointegration process, thereby reducing treatment time by 1–3 months [23].

Nanoparticles are incorporated into toothpaste formulations to alleviate dentin hypersensitivity, control halitosis, regulate the growth of both beneficial and harmful microorganisms, and serve various other oral health applications [21]. Nano-delivery systems used as an adjunctive therapy for periodontitis treatment can be classified into three primary approaches: antibacterial therapy, immunomodulatory therapy, and periodontal tissue regeneration. The high surface-to-volume ratio of nanoparticles enables efficient drug loading or combination therapies, enhancing treatment effectiveness by targeting multiple aspects of periodontitis management. For instance, metal nanoparticles such as silver and gold can directly sterilize or improve drug solubility in water, facilitating better transport to biofilm bacteria. When combined with photodynamic therapy, they can also help overcome bacterial resistance [21].

Various types of nanoparticles can also deliver natural active compounds, such as quercetin, baicalin, and caffeic acid phenethyl ester (CAPE), into periodontal pockets. These agents may help regulate excessive host immune responses or support the repair of damaged periodontal tissues [24,25,26]. For example, Tian et al. applied baicalin-loaded mesoporous nanoparticles to create a synergistic nanotherapeutic strategy for treating periodontitis, targeting both antimicrobial and immunomodulatory effects [27]. The development of novel nanoparticle delivery systems, including biomimetic nanoparticles derived from cell membranes and exosomes, has the potential to broaden therapeutic strategies for using nanoparticles in periodontitis treatment. This review will examine future challenges and research directions for applying nanotechnology in the non-surgical management of periodontitis as a strategy modulating the host’s response. The main characteristics of some nanoparticles are presented in Table 1.

### 2.1. Antimicrobial Nanotherapeutic Strategies

The use of antimicrobial therapy in periodontitis treatment remains a topic of ongoing debate among researchers. Antibiotics have long been a key adjunctive strategy in periodontitis management. However, the use of high doses of antibiotics for more than 10 days often leads to issues such as microbial resistance, which diminishes their long-term therapeutic effectiveness [8]. In recent decades, nanoparticle delivery systems have been engineered to address antibiotic resistance, functioning as independent and effective antimicrobial agents in adjunctive therapy for periodontitis treatment. Furthermore, nanomaterials or nanoparticle delivery systems loaded with photosensitizers can enhance antibacterial effects through photothermal therapy (PTT) or photodynamic therapy (PDT) [28].

### 2.2. Antibiotic Nano-Antibacterial Agents

It is well known that tetracycline antibiotics inhibit protein synthesis by binding to the 30S ribosomal subunit of microorganisms. On the other hand, quinolone antibiotics, such as ciprofloxacin hydrochloride, interfere with DNA synthesis by inhibiting gyrase activity, leading to irreversible damage to bacterial chromosomes [29,30]. Applying nanomaterials as carriers for antibiotics significantly reduces the required medicament dosage, the frequency of its application, and the risk of bacterial resistance, making it a highly effective strategy for treating periodontitis. For instance, minocycline-loaded nanoparticle delivery systems (MIN-NPs) can penetrate deep into periodontal pockets due to their small size, thereby enhancing the antimicrobial effect of locally applied antibiotics. MIN-NPs offer sustained drug release, reducing the need for frequent applications and preventing rapid drug release within the periodontal pocket. Pharmacokinetic studies show that MIN-NPs have a longer retention time in the gingival sulcus compared to minocycline hydrochloride ointment or solution [31]. The concentration of MIN-NPs in the gingival sulcus gradually decreases, maintaining an effective minimum concentration (1.28 μg/mL) for up to 12 days. Similarly, moxifloxacin hydrochloride has been incorporated into nano-systems to enhance its efficacy in periodontitis treatment. Beg et al. [32] used moxifloxacin hydrochloride-loaded nanoparticles (MOX-PLGA), and in vitro release profiles revealed that nanoparticles within an in situ gel resulted in a much slower drug release, with only 20% of the drug released in the first 12 h. This contrasts with the profiles of the group using only the in situ gel, indicating that the gel significantly slows down the antibiotic’s release [32].

### 2.3. Metallic Nano-Antibacterial Agents

The development of metallic nanomaterials has shown new possibilities for antibacterial therapy. Among these materials, gold, silver, titanium dioxide, and zinc oxide (ZnO) have demonstrated significant potential with their antimicrobial properties. Gold nanoparticles (Au-NPs) offer unique physical effects compared to traditional antibiotics. These include their ability to interact directly with the phospholipid bilayer of microbial cell membranes, bind to cytoplasmic proteins, and generate reactive oxygen species (ROS), thereby exerting strong antibacterial effects. Research shows that the growth of Fusobacterium nucleatum is significantly reduced when it is exposed to gold nanoparticles (Au-NPs) at a concentration of 0.2 mM, compromising the integrity of the bacterial cell wall. This suggests that the Au-NP-induced disruption of membrane potential is critical for the energy metabolism of Fusobacterium nucleatum [33,34]. The membrane potential disruption indicates that Au-NPs inhibit bacterial growth by depolarizing the bacterial membrane. Additionally, ROS levels were found to increase following Au-NP exposure. It has also been shown that microorganisms struggle to develop resistance to Au-NPs.

The antibacterial activity of ZnO nanoparticles arises from electrostatic interactions with bacterial cell walls, the adsorption of Zn^2+^ ions onto bacterial surfaces leading to changes in membrane permeability, the generation of reactive oxygen species (ROS), and other antimicrobial actions. Münchow et al. incorporated ZnO nanoparticles into polylactic acid fibrous membranes. Agar plate tests revealed that the ZnO-loaded membranes effectively suppressed the growth of some microorganisms such as *Porphyromonas gingivalis* and *Fusobacterium nucleatum*, with inhibition zones measuring between 6 and 15 mm, demonstrating significant antibacterial potential [35,36,37]. Additionally, the ZnO-loaded polymer film exhibited antibacterial properties while also stimulating reactive oxygen species (ROS) production, boosting cell proliferation, and accelerating wound healing through growth factor activation. These findings indicate that ZnO-loaded nanoparticles have significant potential regarding the treatment of periodontitis [38].

Some researchers hypothesized that incorporating silver nanoparticles (AgNPs) can improve the anti-inflammatory effectiveness of hybrid biomaterials. Wang et al. designed a three-dimensional hybrid material integrating extracellular matrix components, including collagen, chondroitin sulfate, and fibronectin, combined with AgNPs. In vivo studies showed that the hybrid material supported cell and blood vessel infiltration while promoting the synthesis of a new extracellular matrix. Notably, the antibacterial properties of the silver nanoparticles persisted after they were embedded in the polymer network, successfully inhibiting the growth of *Fusobacterium nucleatum* and *Porphyromonas gingivalis*. These results suggest that the addition of silver nanoparticles considerably enhances the biomaterial’s anti-inflammatory effects [39].

**Figure 1:**
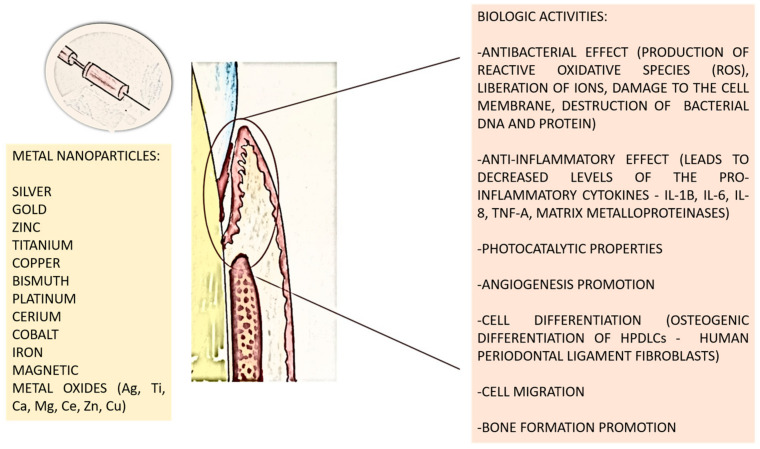
Effects of the most used metal nanoparticles in periodontology.

Steckiewicz and colleagues developed AgNPs combined with chlorhexidine (AgNPs-CHL) or metronidazole (AgNPs-PEG-MET). AgNPs-CHL exhibited greater antibacterial efficacy compared to AgNPs-PEG-MET, though with higher cytotoxicity. Despite this, both groups demonstrated beneficial properties at non-toxic concentrations, including reduced levels of pro-inflammatory cytokines like IL-1β, IL-6, IL-8, and TNF-α, as well as matrix metalloproteinases MMP-3 and MMP-8 [40]. Figure 1 illustrates some essential metal nanoparticles and their biological actions.

ZIF-8 (zeolitic imidazole framework-8)—which involves the binding of zinc ions (Zn^2+^) with imidazolate (Im) ligands—has shown high biological activity, with the extended release of Zn^2+^. The significant effects of ZIF-8 include stimulating anti-inflammatory cytokine production, inhibiting pro-inflammatory cytokines, exhibiting antibacterial properties against *Porphyromonas gingivalis*, and promoting extracellular matrix mineralization, as well as regulating the expression of osteogenic genes and proteins [41].

Other organic nanoparticles, such as dendrimers, chitosan, Poly Lactic-Co-Glycolic Acid (PLGA), ZIF-8, and liposomes, are also highlighted in the literature as potential carriers with active properties. For example, chitosan, a biopolymer, is known for its biocompatibility, biodegradability, and antibacterial and antioxidant effects [42,43,44,45,46]. The data are summarized in Table 2.

Research by Hu et al. showed that nanoparticles made from quaternary chitosan, liposomes, and doxycycline have an affinity for fibroblasts and periodontal ligament cells, inhibit biofilm growth, and prevent alveolar bone resorption [47].

### 2.4. Application of Photothermal and Photodynamic Therapy

Photothermal therapy (PTT) and photodynamic therapy (PDT) are also utilized in the antimicrobial treatment of periodontitis. In comparison to traditional antibiotic treatments, photothermal therapy (PTT) and photodynamic therapy (PDT) have a lower risk of inducing resistance in microorganisms linked to periodontitis. Furthermore, the light source wavelength used in photothermal antimicrobial therapy can penetrate deeper into tissues than other light-based treatments, allowing it to reach the deeper layers of periodontal tissues more effectively. Photothermal antimicrobial therapy works by using an appropriate photothermal material to destroy microorganisms through heat generated during irradiation. For effective results, the photothermal material must respond to near-infrared light [48,49]. The antimicrobial effect of photodynamic therapy (PDT) involves the use of a photosensitizing agent that produces reactive oxygen species (ROS) when exposed to light. This leads to the fast oxidation cascade of lipids in bacterial membranes, ultimately destroying the bacteria. Chlorin e6, a widely used photosensitizer in PDT, is favored for its strong tissue penetration, excellent biocompatibility, and high singlet oxygen yield [50].

Xu et al. [51] proposed an injectable sodium alginate hydrogel composite (CTP-SA), combined with copper dioxide and coated with polydopamine-modified titanium dioxide (TiO2@PDA), designed for guided tissue regeneration (GTR). After injection, CTP-SA transitions from liquid to solid, filling the defect site and adapting to its shape. Additionally, CTP-SA demonstrates antibacterial effectiveness when exposed to blue light (BL). The generation of reactive oxygen species (ROS) under BL excitation accelerates the oxidation of Cu^+^ to Cu^2+^. The photothermal reaction of CTP-SA under near-infrared (NIR) light, combined with its bioactive properties, enhances the osteogenic effect of the hydrogel. In conclusion, through the utilization of dual-light modulation (BL and NIR), CTP-SA achieves both antimicrobial and osteogenic effects, supporting personalized guided tissue regeneration (GTR) procedures. In a separate study, Hu et al. developed an NIR-responsive delivery system based on carvacrol (CA)—UCNPs@mSiO2-CA—which responds to BL. When exposed to NIR light, this system demonstrates antimicrobial, anti-inflammatory, and immunomodulatory effects due to the combined action of CA and the converted BL, influencing key signaling pathways such asMAPK, TNF, and IL-17 [52].

### 2.5. Immunomodulatory Nanotherapeutic Strategies: Macrophage Polarization Remodeling

In periodontitis, microorganisms and their byproducts trigger the host’s immune response. The excessive activation of the innate immune system frequently results in severe inflammation and the disruption of homeostasis, exacerbating existing periodontal lesions. The majority of the tissue damage in periodontitis is attributed more to the host’s immune response than to direct microbial action. Given the immune system’s role in periodontitis progression, researchers have focused on modulating macrophage activity to control inflammation and promote tissue regeneration. Numerous nano-systems have been developed to regulate immune cell function and inflammation-related cytokines, yielding promising therapeutic outcomes both in vitro and in vivo. Some essential blood cells, such as macrophages, which act as key immune cells, serve as the first line of defense against microbial invasion. Influenced by various factors, they can differentiate into different phenotypes, such as M1 and M2, both of which play a role in regulating the host’s immune response. M1 macrophages release cytokines like IL-6 and TNF-α, which trigger inflammatory reactions. On the other hand, M2 macrophages can be further categorized into subtypes, including alternatively activated macrophages (M2a), type 2 macrophages (M2b), deactivated macrophages (M2c), and M2-like macrophages (M2d), based on the specific stimuli and transcriptional factors involved [53,54].

M2a macrophages are induced by IL-4 and IL-13 and secrete factors such as TGF-β, insulin-like growth factor, and fibronectin, all of which contribute to tissue repair. M2b macrophages express and release substantial amounts of the anti-inflammatory cytokine IL-10 while producing low levels of IL-12. M2c macrophages, induced by IL-10, exhibit strong anti-inflammatory activity by releasing large quantities of IL-10. M2d macrophages, also known as tumor-associated macrophages, possess unique characteristics linked to tumor environments.

Shi et al. [55] developed resveratrol-loaded liposomes (Lipo-RSV) to promote macrophage polarization from M1 to M2. Treatment with Lipo-RSV resulted in a 14% increase in the proportion of M2-like macrophage subpopulations, while decreasing M1-like subpopulations by 8%. Mechanistically, Lipo-RSV inhibited the phosphorylation of STAT1 (signal transducer and activator of transcription) and promoted the phosphorylation of STAT3 in bone marrow-derived macrophages. STAT proteins play a crucial role in various aspects of the immune response, including cell proliferation, apoptosis, and differentiation. Furthermore, Lipo-RSV significantly decreased the levels of pro-inflammatory cytokines (IL-1β, IL-6, TNF-α, and IL-12) and reactive oxygen species (ROS), while substantially increasing the level of the anti-inflammatory cytokine IL-10. These findings suggest that the Lipo-RSV method holds promise as a potential treatment for periodontitis by regulating macrophage polarization from M1 to M2 [56].

## 3. Immunomodulatory and Regenerative Therapy for Periodontium

As previously mentioned, immune cells such as M1 macrophages, Th1 cells, Th17 cells, some pro-inflammatory cytokines, reactive oxygen species (ROS), chemokines, and other immune mediators have the potential to damage the periodontal tissues either by direct or indirect mechanisms. For example, Th17 cells are involved in the synthesis of IL-17, which in turn regulates the expression of mRNA for MMP-1 and MMP-3 in the periodontal structures. This results in collagen and proteoglycan breakdown, resulting in the destruction of periodontal tissues [57]. M1 line macrophages release various pro-inflammatory cytokines, such as TNF-α, IL-1β, IL-6, IL-12, and IL-23. TNF-α regulates the expression of RUNX2 (a protein that plays a crucial role in osteoblast differentiation and bone morphogenesis) in osteoblasts, and it increases the expression of RANKL in osteoclast precursors and osteoblasts, contributing to periodontal destruction [58,59]. RUNX2 is essential for osteoblast differentiation, and ALP and COL-I are key indicators of bone formation. Interleukin-1β enhances the production of enzymes like collagenase in osteoblasts and promotes the degradation processes of collagen in the periodontium. Interleukin-6 increases RANKL expression in osteoblasts, inducing the differentiation of osteoclasts and the resorption of alveolar bone. A synergistic approach that combines immunomodulation and periodontal regeneration through nano-drugs is anticipated to greatly enhance therapeutic outcomes in the treatment of periodontitis [60]. In addition to the proven antibacterial properties of gold nanoparticles (Au NPs), they have been shown to successfully modulate macrophage phenotypes and reduce osteoclast activity, making them highly versatile nanoparticles. Au NPs reduce mRNA levels of factors associated with M1 macrophages (TNF-α, IL-6) while promoting factors linked to M2 macrophages (Arg-1, IL-10, and TGF-β). Specifically, after treatment with Au NP-loaded nanoparticles, M2-type macrophages facilitate the osteogenic differentiation of human periodontal ligament stem cells (PDLSCs) [61]. The transcription levels of mRNA for osteogenic factors such as ALP (alkaline phosphatase) and COL-I (type I collagen) in human PDLSCs are elevated in macrophages modulated by nanoparticles toward the M2 phenotype. Similarly, Au NP-modulated M2 macrophages support the formation of mineralization sites in human PDLSCs. Moreover, the intracellular RANKL/OPG (osteoprotegerin) ratio is reduced, suggesting decreased osteoclastogenic activity [62].

## 4. Challenges and Future Perspectives

The human oral cavity harbors approximately 800 species of microorganisms. The hard and soft oral structures provide areas for these species to colonize, forming an ecosystem. *Porphyromonas gingivalis* is considered a key pathogen in the development of periodontitis with complex microbial interactions. Additionally, many pathogens such as *Aggregatibacter actinomycetemcomitans*, *Tannerella forsythia*, *Treponema denticola*, and *Fusobacterium nucleatum* are also associated with the progression of periodontal disease. Most current nano-antibacterial treatment strategies, such as antibiotics or photodynamic therapy (PDT), focus on combating periodontitis by reducing all bacteria in the periodontium. However, studies have shown that certain probiotics, like *Lactobacillus* and *Streptococcus dentisani*, can neutralize or inhibit factors that contribute to periodontal disease progression. The potential mechanisms of *Lactobacillus* include pathogen suppression, the competitive inhibition of pathogenic bacterial adhesion, and combating bacterial biofilms. Wang et al. demonstrated that a cell-free supernatant prepared from *Lactobacillus curvatus* reduced TNF-α, IL-6, and cyclooxygenase-2 gene expression in *Porphyromonas gingivalis* LPS-stimulated human gingival fibroblasts, offering new therapeutic strategies for preventing periodontitis [63,64,65,66,67,68,69]. *Streptococcus dentisani* inhibits the colonization of *Porphyromonas gingivalis* and *Fusobacterium nucleatum* in human gingival fibroblasts and reduces the production of pro-inflammatory cytokines by these pathogens. Therefore, targeted nano-delivery systems or biomimetic approaches can be developed to deliver selective antimicrobial effects, preserving beneficial bacteria and normal tissue cells. The future of nano-based antimicrobial therapy lies in restoring oral microbiota homeostasis, rather than indiscriminately eliminating all microorganisms, including those essential for a healthy oral environment. In periodontal lesions, overactive macrophages or certain T cells produce excessive reactive oxygen species (ROS), which trigger inflammatory pathways and osteoclast maturation, ultimately resulting in the destruction of periodontal tissues and bone. Current nanotherapeutic strategies focus on eliminating ROS as end-products. Several studies have shown that low levels of ROS can help preserve the regenerative potential of periodontal stem cells. Researchers are investigating ways to maintain ROS levels in periodontal tissues at a controlled, low threshold to support tissue regeneration while allowing the products to maintain their antibacterial properties without triggering an immune response or oxidative stress [68,69].

## 5. Therapeutic Approaches for Periodontitis and Related Systemic Diseases

Periodontitis has been linked to various inflammatory, autoimmune, and malignant diseases, including diabetes, rheumatoid arthritis, Alzheimer’s disease, hypertension, inflammatory bowel disease, and cancer [70,71,72]. Given these associations, nano-delivery systems hold promising potential for treating periodontitis in patients with comorbidities. A reciprocal relationship between periodontitis and diabetes has been well established [73]. Periodontitis can exacerbate the severity of diabetes and hinder effective blood glucose control [73,74]. Conversely, metabolic disturbances in patients with diabetes result in the excessive production of reactive oxygen species (ROS), which have a destructive impact on alveolar bone. The impairment of periodontal tissues in individuals with both diabetes and periodontitis is more severe than in those with periodontitis alone. Zhao et al. developed an ROS-responsive drug delivery system, co-loaded with doxycycline and metformin, which shows efficacy in treating periodontitis in patients with diabetes [75]. Wang et al. applied injectable nano-hydrogels containing mesoporous silica nanoparticles composed of poly-lactide-ethylene glycol to mimic the mesenchymal stem cells in the periodontal ligament, aiming to stimulate the osteogenic cascade for periodontal bone repair [76]. In the future, nanotherapeutic strategies are expected to offer enhanced opportunities for addressing periodontal-related complications associated with periodontitis. While nano-delivery systems show potential for managing periodontitis in patients with comorbidities, challenges such as nanoparticle safety, regulatory approval, and cost-effectiveness must be addressed before widespread clinical application. Future nanotherapeutic strategies are expected to address local periodontal damage and systemic complications. However, further research is needed to optimize safety, efficacy, and large-scale clinical applications.

## 6. Therapeutic Potential of Natural Active Ingredients in Nanotechnology

Antibiotics and nonsteroidal anti-inflammatory drugs (NSAIDs) present several challenges in the treatment of periodontitis, such as drug resistance, oral and systemic dysbiosis, and gastrointestinal irritation. Recently, natural compounds sourced from nature have garnered significant attention from different investigators. Natural ingredients with demonstrated therapeutic activity in periodontitis include quercetin, resveratrol, baicalin, and curcumin, among others [77]. Quercetin has a defensive effect in periodontitis by scavenging free radicals that damage cells and increase the risk of disease development. Another study found that quercetin reduced alveolar bone loss by inhibiting inflammation in rats with periodontitis [25]. Resveratrol, a polyphenol, has shown potential in modulating the body’s response, aiding in the prevention of diabetes and osteoporosis and promoting cardiovascular health in patients with heart disease. It also suppresses inflammatory processes in the body and is a powerful antioxidant that protects cellular DNA from mutagenic oxidative damage, slowing cellular aging. These qualities make it a promising natural agent for treating periodontitis [78]. Curcumin significantly reduces the expression of TNF-α and IL-6 by inhibiting the phosphorylation of p38, part of the mitogen-activated protein kinase (MAPK) group. p38 is a mediator of signal transduction linked to inflammation, cell cycle regulation, apoptosis, cell differentiation, aging, and tumorigenesis in specific cell types. Evidence suggests that its activation decreases the inflammatory response in macrophages. Curcumin-2.24 has been shown to mitigate alveolar bone loss in vivo, likely due to its ability to inhibit pro-inflammatory cytokines like IL-1β. Additionally, it reduces the activity of gelatinases (MMP-9 and MMP-8) and their precursor forms in periodontal tissues, contributing to its therapeutic effect [79]. Plumbagin, a natural yellow pigment derived from the roots of Plumbago zeylanica, modulates the expression of mRNA for pro-inflammatory cytokines including TNF-α, IL-1β, IL-6, and PGE2 in periodontal structures. It also affects mediators like histamine, bradykinin, and serotonin, helping to prevent the progression of the inflammatory process [80]. Table 3 summarizes key therapeutic mechanisms in periodontitis treatment, highlighting their biological significance and potential clinical applications.

**Figure 2:**
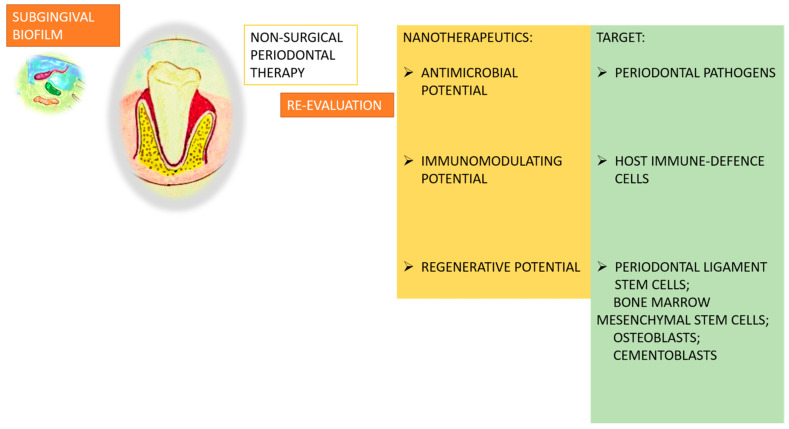
Nanotherapeutics in periodontal treatment plans.

While natural compounds have demonstrated promising experimental results in treating periodontitis, several challenges hinder their clinical application. Many of these bioactive molecules, such as quercetin, resveratrol, baicalin, and curcumin, exhibit poor solubility, low bioavailability, and rapid degradation in physiological environments. Additionally, concerns regarding safety, metabolism, and limited knowledge of their in vivo pharmacokinetics restrict their widespread use in periodontitis therapy. Advanced nano-drug delivery systems offer innovative solutions to overcome these limitations. Nanocarriers, including liposomes, polymeric nanoparticles, dendrimers, and nanomicelles, can enhance the stability, solubility, and controlled release of these natural agents, ensuring prolonged therapeutic effects. For example, encapsulating quercetin in polymeric nanoparticles can improve its bioavailability and target delivery to periodontal tissues, maximizing its antioxidant and anti-inflammatory effects. Similarly, resveratrol-loaded liposomes (Lipo-RSV) have been shown to modulate immune responses and support tissue regeneration by shifting macrophage polarization from M1 (pro-inflammatory) to M2 (anti-inflammatory) phenotypes. By integrating nanotechnology with natural bioactive molecules, future periodontitis therapies may achieve greater precision, reduced side effects, and enhanced efficacy, ultimately paving the way for more biocompatible and sustainable treatment options. Their growing utilization underscores the potential for future clinical applications, as illustrated in Figure 2.

## 7. Discussion

The use of nanoparticles (NPs) raises important concerns regarding their availability, sustainability, recyclability, synthesis, and manufacturing processes. These challenges stem from their size, surface properties, and composition, which influence their biological behavior, potential toxicity, stability, and life cycle. For instance, some nanoparticles, such as titanium dioxide (TiO_2_), remain stable when exposed to acidic recycling processes, while others, like zinc oxide (ZnO), dissolve completely. Carbon-based nanoparticles can degrade over time, further complicating their long-term stability. Beyond stability, NPs may have negative effects on cells and cellular structures, including the cell membrane, mitochondria, lysosomes, Golgi apparatus, and nucleus. These interactions can lead to DNA damage, enzyme dysfunction, lipid peroxidation, and protein oxidation, which may compromise cellular function. Given these potential risks, there is a growing need for clear regulatory guidelines to ensure the safe application of nanoparticles in medical and environmental settings. The stability of nanoparticles is also influenced by their interactions with biological environments, such as blood vessels and immune system cells. Lipid-based and polymer-based nanoparticles, in particular, are highly susceptible to instability and aggregation both in circulation and during storage. To counteract this, researchers have explored various strategies to enhance nanoparticle stability, including surface modifications with lipids and lyophilization techniques to improve storage and transport before clinical use. After administration, the distribution of nanoparticles is affected by their shape and size. These physical properties determine their ability to move through tissues, extravasate from blood vessels, and localize in specific areas. Studies suggest that non-spherical nanoparticles, such as those with rod, discoid, and elliptical shapes, achieve better tissue margination compared to spherical nanoparticles, making them more effective for targeted drug delivery [81].

A key challenge in nanoparticle therapy is finding the right balance between stability and effective intracellular delivery. Once in the body, nanoparticles interact with serum proteins and lipids, which can alter their chemical and physical properties. This, in turn, affects their distribution, stability, and ability to reach target tissues. Additionally, these interactions influence how nanoparticles are recognized and cleared by immune system cells, such as phagocytes and dendritic cells. Research indicates that cationic nanoparticles are cleared most rapidly, followed by anionic nanoparticles. Neutral or slightly negatively charged nanoparticles, on the other hand, have the longest circulation half-life, making them more suitable for sustained therapeutic effects [82]. Despite their promising applications, nanoparticles pose potential risks, including hypersensitivity reactions, aggregation, and toxicity. Metal-based nanoparticles have been linked to immune responses and allergic reactions, raising concerns about their biocompatibility. Instability within biological systems remains a significant challenge, as nanoparticles may shift from their intended target sites. Some nanoparticles undergo enzymatic degradation, which can alter their effectiveness. Others suffer from slow absorption and uncontrolled release, reducing their efficiency in drug delivery. Regulatory and ethical concerns further complicate the widespread use of nanoparticles. Currently, there is limited knowledge regarding their long-term effects, and many aspects of their safety remain unexplored. The lack of comprehensive studies and standardized guidelines underscores the need for further research. Future in vivo studies will be crucial in understanding how nanoparticles function within living systems, ensuring that their use is both safe and effective. Moving forward, advancements in nanoparticle design should focus on improving biocompatibility and addressing potential health risks. Strengthening regulatory frameworks and conducting long-term safety assessments will be essential for integrating nanoparticles into clinical applications while minimizing potential risks [83]. Despite considerable research and promising potential, the clinical use of nanomedicines remains significantly below expectations. This gap is primarily due to discrepancies between animal and human studies, which arise from a limited understanding of physiological and pathological differences across species. Moreover, the heterogeneity within patient populations further impedes clinical success, as there is insufficient research on how nanomedicine interacts within specific patient subgroups. As a result, only a few nanomedicines are approved as first-line treatments, with many demonstrating efficacy only in select patient groups. This issue can be attributed to the underexplored variability in disease biology and patient demographics, which influence nanoparticle distribution and function in diseased tissues. Early generations of nanoparticles (NPs) faced substantial challenges in achieving effective biological delivery [83]. However, recent advances in nanoparticle synthesis have led to the development of more sophisticated structures and targeting agents, significantly improving drug delivery. These modern NPs now function as multifunctional systems, including combination therapies, to enhance therapeutic efficacy and overcome drug resistance. Polymeric NPs show great promise as drug delivery systems due to their biodegradability, water solubility, biocompatibility, biomimetic properties, and storage stability. Their surfaces can be modified to enhance the targeted delivery of drugs, proteins, and genes, making them valuable in oncology, gene therapy, and diagnostics. However, challenges remain, such as particle aggregation and potential toxicity. Currently, only a few polymeric nanomedicines have received FDA approval, though many are still undergoing clinical trials. Some inorganic NPs, despite their excellent biocompatibility, have limited clinical applications due to poor solubility and toxicity concerns, particularly when heavy metals are involved [83]. The clinical application of nanoparticle-based anesthesia also presents several challenges, detailed below.

Rapid clearance: Nanoparticles, particularly rigid or cationic ones, are rapidly eliminated from the bloodstream, while neutral or weakly negative NPs tend to have longer circulation times. NPs can activate the mononuclear phagocytic system (MPS), triggering immune responses that may result in inflammation or tissue damage by causing immune system interactions. The size and shape of NPs significantly influence immune responses and can lead to varying degrees of inflammation. Certain polyethylene glycol (PEG)-modified NPs have been linked to severe allergic reactions or anaphylaxis in some patients during clinical trials [83].

In conclusion, while nanomedicine continues to make progress, significant barriers—including patient variability, safety concerns, and regulatory challenges—must be addressed to enhance clinical translation. Future research should focus on refining nanoparticle design, improving biocompatibility, and conducting extensive clinical trials to ensure the safe and effective application of nanomedicine in clinical settings [83]. At this stage, concerns primarily revolve around the potential oral toxicity of nanomaterials and their effects on organ health. However, clinical studies conducted so far indicate that the risks associated with nanomaterials are minimal and manageable. To maximize their benefits, continued innovation in nano-delivery system design is essential. It is also crucial to acknowledge that most studies have been conducted in vitro. More extensive in vivo and clinical research will be required to fully understand the potential of nanotherapeutic technologies, ensuring their safe and effective application in periodontal disease treatment [81,82,83,84].

As stated by Wang et al., the nanomaterials used in periodontal tissue must comply with biosafety standards to prevent any damage to healthy tissue structures. To effectively inhibit bacterial growth, whether through the nanoparticles themselves or the drugs they carry, the released drug concentration must exceed the minimum inhibitory concentration. Therefore, nanomaterials must regulate the rate and concentration of drug release to maintain a consistent antimicrobial effect throughout the treatment period [39]. Currently, only a limited number of clinical studies have explored the use of nanoparticles (NPs) in treating periodontitis, with most research still in the preclinical phase. This suggests that certain challenges are preventing the successful translation of academic findings into clinical applications. In conclusion, overcoming these obstacles is essential to advancing the clinical implementation of NPs for effective periodontitis treatment [39]. While many studies have examined the short-term toxicity of nanoparticles (NPs), there is still limited knowledge about their accumulation in vital organs or tissues, which may lead to unexpected toxic effects. Therefore, understanding the long-term behavior of NPs—including their absorption, distribution, metabolism, and excretion—along with monitoring potential side effects remains a significant challenge. In conclusion, addressing these concerns is crucial to ensuring the safe and effective use of NPs in medical applications [39].

Nanomedicine has the potential to revolutionize dentistry and periodontal therapy by providing more precise, effective, and minimally invasive treatment options. As nanotechnology continues to advance, it is anticipated to reshape how dental professionals prevent, diagnose, and manage various oral diseases, particularly periodontal conditions. However, for its successful clinical implementation, critical challenges such as biocompatibility, targeted delivery, stability, and cost-effectiveness must be addressed [85]. Future research should focus on overcoming these barriers by optimizing nanomaterial formulations, enhancing long-term safety profiles, and developing cost-efficient manufacturing processes. Additionally, conducting extensive clinical trials will be essential to validate their efficacy and ensure widespread adoption in dental practice.

In the realm of nanotechnology for periodontal therapy, several critical challenges must be overcome to enable its integration into clinical practice. These include biocompatibility and safety concerns, regulatory hurdles, ethical considerations, and substantial costs. Ensuring the safety of nanomaterials in human applications requires rigorous preclinical and clinical trials, as research has shown that their toxicity can be influenced by factors such as shape, size, and chemical composition. Another major obstacle stems from the evolving legal landscape governing nanotechnology-based treatments. For clinical adoption, these innovations must adhere to stringent safety and efficacy standards, necessitating robust regulatory frameworks and standardized nanomaterial characteristics. Before reaching the market and becoming available to patients, these materials must secure FDA (Food and Drug Administration) approval and comply with predefined criteria. Moreover, ethical concerns surrounding patient consent and the long-term implications of nanomaterials present additional challenges. The unpredictable nature of potential risks, including effects on future generations, raises crucial ethical debates that require public engagement. Finally, integrating nanotechnology into periodontal therapy entails considerable financial investment due to the need for advanced technology and specialized equipment. High research and development expenditures, manufacturing costs, and market accessibility constraints pose significant financial barriers for both researchers and practitioners [85].

Despite the promising potential of nanotechnology and nanomaterials in biomedical applications, several significant challenges impede their transition from laboratory research to clinical practice. A primary obstacle is the absence of standardized protocols for designing nanomaterials with precise composition, structural parameters, and physicochemical properties, making it difficult to optimize their in vivo behavior and performance. Additionally, biosafety concerns and potential adverse effects, such as immune reactions triggered by nanomaterial exposure, remain critical issues. Controlling their biodegradation is another challenge, as biodistribution and pharmacokinetics are influenced by factors like size, shape, and surface chemistry, while achieving uniform production remains complex. Moreover, for effective local drug delivery, the precise regulation of the biodegradation rate is essential [86,87,88,89]. Overall, while nanomaterials offer significant potential for tissue regeneration and periodontal therapy, addressing their challenges is crucial. Future research should prioritize the development of standardized fabrication methods, the optimization of surface chemistry for enhanced biocompatibility, and improved control over biodegradation and biodistribution. Overcoming these obstacles will be essential to fully realizing the clinical potential of nanotechnology.

Effective drug delivery systems are essential for releasing therapeutic agents—such as growth factors, osteogenic drugs, periodontal ligament stem cells, and adhesion factors—at the wound site to enhance treatment outcomes [90]. Future research should focus on personalizing local drug delivery systems to refine clinical protocols and improve the efficacy of periodontal therapy. While nanoparticles (NPs) offer unique advantages, their properties also present potential health and environmental risks, necessitating responsible use. For example, the small size of TiO_2_ increases its photoreactivity, enabling microorganism oxidation but also causing unintended oxidative damage to other organisms, such as fish. Although efforts have been made to identify, classify, and mitigate these risks, comprehensive assessments of long-term consequences remain scarce, requiring further investigation. Despite significant U.S. government funding for nanotechnology in 2018, only 0.6% was allocated to the Environmental Protection Agency, underscoring the limited emphasis on environmental safety. Additionally, the complexity and heterogeneity of NPs complicate their overall evaluation, demanding interdisciplinary collaboration across fields such as physics, environmental engineering, chemistry, biology, and toxicology [81].

Nanotechnology holds great promise for periodontal therapy, but several challenges must be addressed for its successful clinical integration. Key obstructions include biocompatibility and safety concerns, regulatory approval complexities, ethical considerations, and high costs associated with research and specialized equipment. The lack of standardized protocols for nanomaterial design, along with biosafety issues such as immune reactions and biodegradation control, further complicates its transition from research to practice. Additionally, achieving uniform production and precise drug delivery remains difficult. Future research should focus on standardizing fabrication methods, optimizing surface chemistry for improved biocompatibility, and refining local drug delivery systems to enhance treatment efficacy. However, the unique properties that make nanoparticles effective also pose environmental and health risks, such as oxidative damage from materials like TiO_2_. Despite efforts to assess and mitigate these risks, long-term consequences remain insufficiently studied, partly due to limited funding for environmental safety. Addressing these challenges requires interdisciplinary collaboration across fields like physics, environmental engineering, chemistry, biology, and toxicology to ensure the responsible and effective use of nanotechnology in clinical applications.

## 8. Conclusions

Thorough investigations into the pathogenesis of periodontitis and the development of nano-delivery systems for antibacterial agents have led to significant advancements in therapeutic strategies. These innovations focus on modulating the host’s immune response, ultimately improving treatment outcomes. The unique physicochemical properties and targeted actions of nano-delivery systems allow for the effective use of various medications in periodontitis treatment, enhancing their stability, bioavailability, and therapeutic efficacy. This article has summarized the nano-systems and strategies that have been developed and clinically tested for the non-surgical treatment of periodontitis. These advancements offer valuable insights and inspiration for future treatment approaches, as well as the continued innovation of nano-systems designed for clinical applications. While nanotherapeutics have shown considerable promise in preclinical research, further clinical studies are needed to fully assess their long-term safety and efficacy. Looking ahead, nanotherapeutic approaches are expected to provide new opportunities for better modulation of the body’s response to periodontitis. This could lead to improved treatment options for patients while addressing broader public health concerns. An increasing body of evidence links periodontitis to systemic conditions such as diabetes, Alzheimer’s disease, rheumatoid arthritis, colitis, and cancer, highlighting the need for advanced therapeutic strategies. Future research should prioritize the standardization of nano-delivery system fabrication, ensuring consistency in composition, structural properties, and biodegradation rates to optimize therapeutic outcomes. Additionally, addressing regulatory and ethical challenges will be crucial for translating these innovations into widespread clinical use. Given the potential health and environmental risks associated with nanomaterials, interdisciplinary collaboration among researchers in medicine, toxicology, environmental science, and material engineering is essential to develop safer, more effective treatments.

## Figures and Tables

**Table 1:** Nanoparticles’ characteristics.

Type of Nanoparticle	Origin	Classification	Properties	Action
Phenethyl Caffeate Ester (CAPE)	Propolis	Belongs to phenolic compounds with an ester group	Antioxidant and anti-inflammatory properties	Clears free radicals that cause oxidative stress and cell damage
Quercetin	Vegetables, herbs, grains, and wine	Flavonoid, belonging to the polyphenol class	Antioxidant action	Acts against free radicals, which damage cells and increase the risk of disease
Baicalin	From the mint family (Lamiaceae)	Flavonoid	Immunostimulant, anti-cancer, and antiviral properties	Antioxidant and anti-inflammatory action by suppressing the release of pro-inflammatory cytokines

**Table 2:** Nanoparticles applied in periodontal diseases.

	Nanoparticles	
Inorganic	Organic	Composite
Metals Metal oxides Hydroxyapatite Silicon dioxide UCNPs (crystalline)	Polydopamine Chitosan Dendrimers PGLA ZIF-8 Lysosomes	Hydrogel Nanofibrous Films Membranes

**Table 3:** Nanoparticles as local antimicrobial means in periodontal therapy.

Mechanism	Mean and Media	Application Form	Biodegradability	Trade Name
Protein synthesis inhibitors	- Doxycycline hyclate in poly(dl-lactide) and N-methyl-pyrrolidone	gel	yes	Atridox
	- Minocycline hydrochloride in polymer (PGLA or poly(glycolide-co-dl-lactide)	microspheres	yes	Arestin
	- Minocycline hydrochloride dihydrate in hyetellose magnesium chloride hexahydrate, ammonio methacrylate copolymer, triacetin and glycerol	ointment	yes	Dentomycin
	- Tetracycline hydrochloride in ethylene vinyl acetate	fiber	no	Actisite
	- Piperacillin/Tazobactam in polymeric volatile carrier (hydroalcoholic solution)	film	yes	Gelcide
Bactericidal action	- Chlorhexidine in xanthan-based gel	gel	yes	Chlosite
	- Chlorhexidine gluconate in hydrolyzed gelatin matrix	film	yes	PerioChip
Nucleic acid metabolism interference	- Metronidazole benzoate in glyceryl mono-oleate and triglyceride	gel	yes	Elyzol

## Data Availability

All available data are published in the manuscript.

## Abbreviations

The following abbreviations are used in this manuscript:

NSAIDs	Nonsteroidal anti-inflammatory drugs
PTT	Photothermal therapy
PDT	Photodynamic therapy
MIN-NPs	Minocycline-loaded nanoparticle delivery systems
MOX-PLGA	Moxifloxacin hydrochloride-loaded nanoparticles
ZnO	Zinc oxide
Au-NPs	Gold nanoparticles
ROS	Reactive oxygen species
AgNPs	Silver nanoparticles
AgNPs-CHL	AgNPs combined with chlorhexidine
AgNPs-PEG-MET	AgNPs combined with metronidazole
ZIF-8	Zeolitic imidazole framework-8
PLGA	Poly Lactic-Co-Glycolic Acid
CTP-SA	Sodium alginate hydrogel composite
TiO2@PDA	Polydopamine titanium dioxide
GTR	Guided tissue regeneration
BL	Blue light
NIR	Near-infrared light
CA	Carvacrol
Lipo-RSV	Resveratrol-loaded liposomes
STAT	Signal transducer and activator of transcription
PDLSCs	Periodontal ligament stem cells
